# Indoor Positioning of Low-Cost Narrowband IoT Nodes: Evaluation of a TDoA Approach in a Retail Environment

**DOI:** 10.3390/s22072663

**Published:** 2022-03-30

**Authors:** Daniel Neunteufel, Stefan Grebien, Holger Arthaber

**Affiliations:** 1 Institute of Electrodynamics, Microwave and Circuit Engineering, TU Wien, 1040 Vienna, Austria; holger.arthaber@tuwien.ac.at; 2 Institute of Signal Processing and Speech Communication, Graz University of Technology, 8010 Graz, Austria; grebien.stefan@gmail.com

**Keywords:** indoor localization, positioning, internet of things, time difference of arrival, angle of arrival, received signal strength, software-defined radio

## Abstract

The localization of internet of things (IoT) nodes in indoor scenarios with strong multipath channel components is challenging. All methods using radio signals, such as received signal strength (RSS) or angle of arrival (AoA), are inherently prone to multipath fading. Especially for time of flight (ToF) measurements, the low available transmit bandwidth of the used transceiver hardware is problematic. In our previous work on this topic we showed that wideband signal generation on narrowband low-power transceiver chips is feasible without any changes to existing hardware. Together with a fixed wideband receiving anchor infrastructure, this facilitates time difference of arrival (TDoA) and AoA measurements and allows for localization of the fully asynchronously transmitting nodes. In this paper, we present a measurement campaign using a receiver infrastructure based on software-defined radio (SDR) platforms. This proves the actual usability of the proposed method within the limitations of the bandwidth available in the ISM band at 2.4 GHz. We use the results to analyze the effects of possible anchor placement schemes and scenario geometries. We further demonstrate how this node-to-infrastructure-based localization scheme can be supported by additional node-to-node RSS measurements using a simple clustering approach. In the considered scenario, an overall positioning root-mean-square error (RMSE) of 2.19 m is achieved.

## 1. Introduction

Location awareness has become an important topic in modern internet of things (IoT) applications. An overview of suitable techniques and possible applications is given in [1,2,3]. In the work at hand we address the topic of localization of static IoT nodes communicating in the sub-6 GHz ISM radio bands. We thereby focus on the physical layer, rather than on higher level signal processing methods. Nodes with low-power radio transceiver chips are deployed in high numbers, for example in retail or warehouse environments such as electronic shelf labels (ESLs). The power consumption of such nodes is a critical parameter as a battery lifetime of several years is required. It is therefore beneficial to keep the required processing on the node to a minimum and shift complexity towards the infrastructure. Due to the high lifetime and large number of deployed nodes, it is infeasible to change the node hardware. Localization shall be enabled as an additional feature of the nodes with as little active time as possible and without modifications to the hardware. While the constraints on the node hardware are rather strict, available measurement times are typically high as the considered scenarios are static. Further, the modification of the infrastructure, e.g., access points (AP) for the nodes, is easier and cheaper than replacing a vast number of nodes.

A widely used technique that generally fulfills the stated requirements is the acquisition of node-to-node received signal strength (RSS) measurements, which is supported by most available transceiver chip front-ends. This is a cheap method from a hardware perspective as no additional infrastructure is required, and thus vigorous research activities are ongoing [4,5]. Many measurements can be performed in a short period of time in scenarios with high number of existing nodes. However, due to severe multipath fading of the radio channel, the obtained results tend to be unreliable. Sophisticated algorithms and/or numerous reference nodes at known positions are required to allow for accurate positioning [6]. Furthermore, RSS quantization is often inaccurate and additional hardware features such as automatic gain control (AGC) result in non-monotonic readouts.

The use of time of flight (ToF) techniques is often impeded by the low signaling bandwidth of common low-power node hardware. Typical radio frequency communication protocols used in IoT applications, such as ZigBee, Bluetooth Low Energy (BLE), etc., use data rates around 1 Mbit/s or less [7,8] and signal bandwidths not exceeding 1 MHz. Using such signals for ToF measurements implies that the achievable resolution of multipath components of the radio channel is limited to some 100 m [9], unless combination with other methods are applied [10]. Even with multi-path assisted localization approaches [11], the fundamental limitation due to the signaling bandwidth persists. In general, the Cramér-Rao lower bound (CRLB) on the positioning error variance is inversely proportional to the bandwidth of the transmitted signal [9]. Thus, a higher transmission bandwidth leads to an improved achievable positioning accuracy.

### 1.1. Contribution

In our previous work, we demonstrated a way to overcome the fundamental limitation of ToF-based localization of narrowband transceiver chips [12,13]. The proposed method requires only a firmware update and no changes to existing node hardware. This is achieved by implementing a customized wideband modulation directly in the radio of the chip. As precise time synchronization between infrastructure and transmitting node or among nodes is not feasible, only time difference of arrival (TDoA) can be used. A receiving anchor infrastructure of some kind is required to capture the transmitted waveform. Using multiple coherent receiver channels per infrastructure anchor, allows for additional angle of arrival (AoA) measurements and triangulation. In order to demonstrate the general practicality of the suggested localization scheme, an exemplary realization of a receiver infrastructure was designed based on commercially available software-defined radio (SDR) platforms. This system was used for the localization of more than one thousand test nodes in an operating grocery store.

The limited bandwidth of 80 MHz in the ISM band at 2.4 GHz dictates fundamental limits regarding the achievable localization accuracy in indoor scenarios with dense multipath components (DMC) channels causing severe fading. In this light, exact localization of nodes remains unfeasible. Nevertheless, for certain applications, it can already be sufficient to identify the correct shelf or aisle in which a node is located. Thus, the obtained position measurements are analyzed in that regard.

The implications on the further development and deployment of this localization scheme are discussed, especially the suitable anchor placement based on the localization results, obtained during the presented measurement campaign.

As the used transceiver chips support RSS measurements natively, a basic concept for data fusion is introduced. It is based on node-to-node RSS measurements performed in parallel to the proposed node-to-infrastructure method. This additional information can be used, for example, for simple clustering which has the potential to improve the overall performance.

The addressed research questions are:

- Can commonly used fully-asynchronous narrowband IoT radio transceiver chips be used for ToF-based localization?
- What positioning accuracy can be achieved in real-world scenarios without using any prior information such as scenario geometry or reference nodes at known positions?
- What receiver configurations are beneficial in this regard?
- Given the known limitations of ToF-based techniques at a fixed bandwidth, what are the possible benefits of data fusion with other techniques, namely AoA and RSS?

The key contributions of this work are:

- Proof of concept for the suggested wideband localization scheme on narrowband transceiver chips.
- Demonstration in an operating store under real-world conditions with a focus on the physical layer.
- Analysis of possible anchor placements and requirements of a receiver infrastructure.
- Evaluation of a data fusion approach for node-to-infrastructure wideband TDoA and AoA with narrowband node-to-node RSS measurements.

### 1.2. Related Work

The characteristics of the DMC radio channel in indoor scenarios as assumed in this work are described in the literature [14,15]. Fundamental performance bounds for localization in scenarios as considered here are evaluated in [9,16,17,18,19,20]. The crucial prerequisite for ToF and AoA-based localization is the synchronization of the receiving anchors. In [21], we elaborated a theoretical framework to evaluate the localization performance bounds for a limited time synchronization quality among anchors for TDoA and phase synchronization quality among the anchor channels for AoA calculations. It is based on the performance bound evaluation for array-based positioning in DMC environments [18] and includes a hybrid parameter Bayesian CRLB framework [22] (sct. 1.2.7), to account for anchor synchronization quality. This framework including the signal model was used for the maximum likelihood estimation (MLE) of the transmitter positions and the performance bound calculations used in the work at hand.

For ToF-based localization, there is hardware available (N_ANOTRON_ NLSG0501A–nanoLOC Transceiver TRX) readily supporting IEEE 802.15.4a chirp spread spectrum (CSS). The generated waveforms can be used for indoor localization in principle [23]. However, due to the comparatively high costs per unit, this is not a viable solution for applications with a high number of nodes. For the same reason, the use of basically any orthogonal frequency-division multiplexing (OFDM) modulation scheme is inhibited. A possible approach to localize fully asynchronously non-cooperatively transmitting nodes in a similar but more contained scenario is presented in [24]. There, time synchronization of the receiving anchors is achieved wirelessly by using reference anchors at known positions. Such an approach can be used also in conjunction with nodes transmitting customized wideband signals according to the method suggested by us. Similarly, even though ultra-wideband (UWB) has been already used successfully for indoor localization in the past [17,25,26,27], it is considered infeasible in the given scenario as it is too costly and also requires changes to existing hardware. Still, it might be an option for the required time synchronization of the anchors.

An entirely different approach suitable for simple node hardware is based on frequency domain phase difference of arrival (FD-PDoA) [28,29]. This technique has mainly been used with backscatter modulation techniques such as RFID. In the case of narrowband nodes considered here, a similar approach is conceivable, based on continuous wave (CW) signals transmitted by one and replied equally by another node. This requires a high level of synchronization between pairs of nodes. The resulting protocol overhead and increased air-time might be unsuitable for applications with tight power consumption constraints. Further, the access to IQ-data, required for such an approach, is often unavailable on the used hardware.

### 1.3. Paper Outline

This paper is structured as follows. In Section 2, technical details on the proposed wideband generation scheme and the required receiver infrastructure are outlined. The measurement scenario and the used hardware implementation is described in Section 3. In Section 4, the obtained localization results are discussed, together with an analysis regarding possible anchor placement schemes and possible clustering based on node-to-node RSS measurements. We conclude the paper in Section 5 and give an outlook on future work on this topic in Section 6.

## 2. Localization Scheme

In this section, we present technical details on the suggested localization scheme. The fundamentals of the wideband generation on the node transceiver chips as well as the basic requirements on the receiver infrastructure are presented.

### 2.1. Nodes

**Figure 1:**
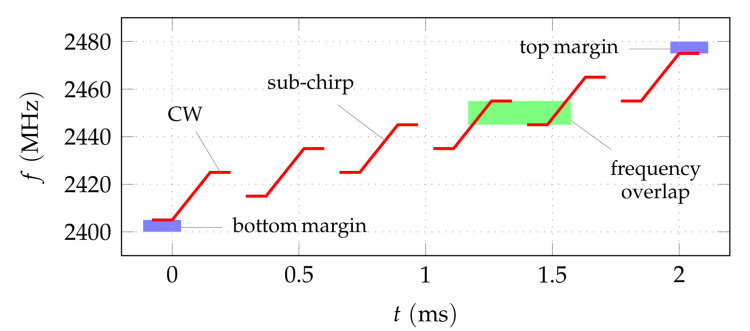
Basic characteristics of the proposed modulation format in the time-frequency plane. Customized chirped waveform generation allows to cover a bandwidth much larger than the typical transmission bandwidth of about 1 MHz. The typical transmitter hardware characteristics limit the achievable chirp-width to about 10 MHz to 12 MHz. By using overlapping sub-chirps, an even larger bandwidth can be covered coherently if the phase relations between the sub-chirps are recovered. Leading and trailing continuous wave (CW) periods allow for the required frequency synchronization between transmitter and receiver. Due to hardware limitations, band margins are required which limits the usable bandwidth within the available frequency band.

The typically used radio front-ends of the deployed transceiver chips, for example the TEXAS INSTRUMENTS CC2510, use a synthesizer based on a phase-locked loop (PLL) [30] for generating the signal carrier frequency. Data are modulated onto a carrier signal at a fixed frequency, using modulation formats such as Gaussian frequency-shift keying (GFSK) or minimum-shift keying (MSK). The modulation is usually encapsuled within the chip and accessible to the developer via an API. Accessing the synthesizer directly allows to extend the possible modulation formats within the given hardware limitations. In [12], we demonstrated exemplarily how frequency chirp modulation is possible on the CC2510 transceiver chip with chirp-widths of up to 12 MHz. The maximum bandwidth achievable in this way is limited by the characteristics of the synthesizer. An extension to a larger bandwidth is possible by concatenating such chirps while changing the range setting of the synthesizer in between these sub-chirps. This inevitably results in loss of phase coherence between the sub-chirps. However, the phase relation between the sub-chirps can be recovered by using an overlap in frequency as seen in Figure 1. The actually usable ranges of the sub-chirps depend on the exact hardware properties of the individual chip. Hence, they must be determined beforehand by means of a self-calibration method. For the same reason, it is not possible in general to choose the exact start and stop frequencies of the sub-chirps freely and, consequently, to cover the available frequency band entirely. The required margins to the band edges reduce the effective bandwidth to about 70 MHz (of the 80 MHz in the ISM band at 2.4 GHz) in the implementation used in this work. Estimating the frequency offset between transmitter and receivers, e.g., by using leading and trailing CW periods together with said self-calibration, allows for a full parameterization of the generated waveform. This chip-specific signal model is used to apply a matched filter to the raw data received by the wideband infrastructure. The application of the matched filter on the chirped waveform makes this method robust with regard to interference from, e.g., Wi-Fi or Bluetooth, which operate in the same frequency band. The output of the matched filter resembles the individual radio channel impulse responses and carries the full information required for transmitter localization. Details on the described method, measured chirp data, and extracted pulses from the CC2510 are presented in [13].

Apart from the CC2510, any transceiver chip with a similar synthesizer is potentially capable of generating the proposed chirped waveform generation in principle. The concrete performance, especially with regard to the width of the sub-chirps, can vary. Tests with different transceiver chips (SILICON LABS EFR32FG22) are ongoing and have indicated a comparable performance so far. Further, as long as the modeling of the generated waveform allows for matched filtering at the receiver side, any customized modulation scheme could be used in a similar manner.

While such transceiver chips can be tweaked to generate and transmit wideband signals in the way described above, it is impossible to increase their receiver bandwidth correspondingly. This means that the proposed method requires a dedicated wideband receiver infrastructure. Nonetheless, a major step towards successful localization is achieved as the use of ToF-based techniques is made feasible without the need for performing any major tasks at the low-power nodes. This shifts the required complexity from nodes to infrastructure and allows to keep existing IoT node hardware.

### 2.2. Infrastructure

Narrowband receivers, possibly identical to the transmitting nodes, can be used for RSS or phase-based localization schemes. This is impossible for the proposed wideband method with chirped transmit signal modulation. Unlike in frequency-modulated continuous-wave (FMCW) radar, which uses similar waveforms and small receiver bandwidths compared to the bandwidth of the actual signal [31] (chp. 2), the transmitter and receiver are neither synchronized in time nor coherent in the sense of a common frequency reference. Thus, a receiver bandwidth and sampling rate sufficiently high to cover the full signal bandwidth at once is required. For the implementation presented here, a (complex) sampling rate of at least 80 MS/s is necessary to fully utilize the available signal bandwidth.

To perform TDoA calculations, multiple time synchronized receiving anchors are required. For AoA measurements, at least two coherent receiver channels per anchor are needed. As a customized radio solution is out of scope of the presented proof of concept, only a limited choice of platforms fulfill these requirements at reasonable costs. While several integrated radio chips such as the LIME MICROSYSTEMS LMS7002M (as deployed for example in the LimeSDR platform) or the ANALOG DEVICES AD9361 are suitable as receiver hardware in principle and might be used in future implementations, the ETTUS RESEARCH/NI Universal Software Radio Peripheral (USRP) X310+TwinRX platform (NI-2945R) offers simple synchronization and an open source driver framework and was thus used in the setup presented in this paper. Details can be found in Section 3.2.1.

The required high sampling rate results in a considerable amount of raw data. It is therefore advantageous to perform pre-processing of the data as described in Section 2.1. In this way, local storage of the raw data is not required and the extracted pulses may be uploaded to remote servers for the application of the actual localization algorithm. The typical amount of data per localization measurement for one node was 3 GB uncompressed raw data and about 100 kB after pre-processing in the implementation presented in this work.

## 3. Test Measurements

In order to verify the functionality of the introduced wideband localization concept of blindly transmitting nodes and a receiving infrastructure, a measurement campaign was conducted. Nodes capable of running a wideband modulation firmware as described in Section 2.1 were distributed as ESLs in an operating grocery store. This scenario is described in Section 3.1. A wideband receiver infrastructure explained in Section 3.2 designed according to the requirements discussed in Section 2.2 was used. A detailed presentation and discussion of the obtained measurement results is given in Section 4.

### 3.1. Measurement Scenario

#### 3.1.1. Environment

The chosen test environment is a grocery store south of Graz, Austria. It is a one-story building with a sales area size of about 800 $m^{2}$ (38×21m). The measurement campaign took place in December 2020. The localization measurements were controlled remotely and performed during and outside of the opening hours. Due to the limited overall time with access to the store, a detailed study of the differences between peak and off-peak hours was not feasible. Therefore, continued work on this topic currently includes investigations in this regard, as well as controlled studies of a varying radio channel, e.g., due to people moving within the scenario.

The operating frequency band was the ISM band at 2.4 GHz with an available bandwidth of 80 MHz. Accompanying node-to-node RSS measurements were conducted simultaneously. Details are not covered in this work but are analogous to the measurement campaign described in [6].

**Figure 2:**
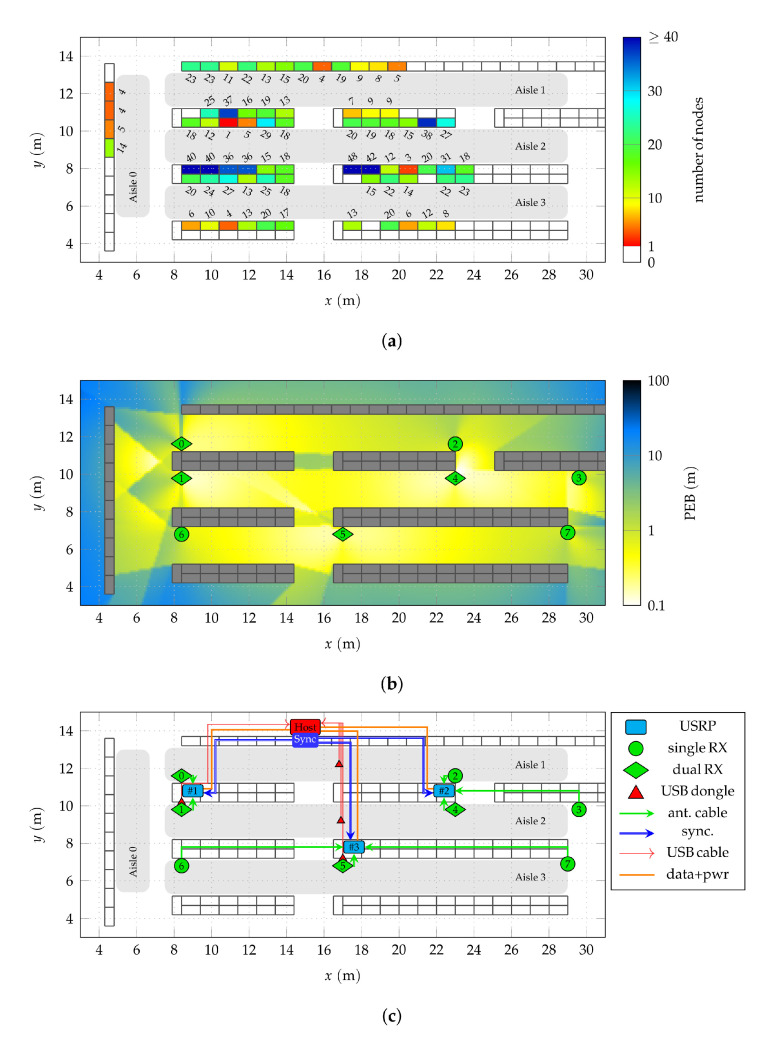
Floorplan of the covered area highlighting different aspects of the scenario, including the aisles labeled 0 to 3. (**a**) Number of nodes per shelf. There are 71 populated shelves with 1265 nodes in total. (**b**) position error bound (PEB) for the used anchor placement in the covered area. (**c**) Locations of the wideband localization infrastructure components and cabling. The three USRP radio platforms are labeled #1 to #3, the eight anchors l=0,⋯,7.

**Figure 3:**
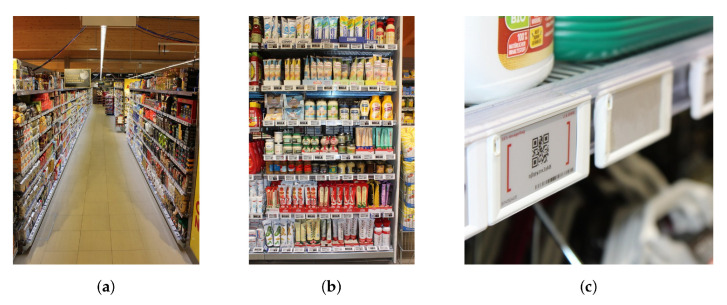
Grocery store test environment. (**a**) Aisle 1 with anchor 0 in the foreground. (**b**) Single shelf at x=13.5 m, y=10 m. (**c**) Transmitter node closeup.

A part of the store was equipped with a wideband receiver infrastructure. The covered area is shown in Figure 2 and all following floorplan schematics. This area comprises three parallel aisles in lateral *x*-direction, labeled 1 to 3, as well as one aisle orthogonal to the others, labeled aisle 0. If relevant, the aisles are indicated in the floorplans. The width of the parallel aisles is 2 m, separated by rows of shelves with a depth of 1 m (2×0.5m back-to-back) and a height of about 2.4 m. Figure 3a shows aisle 1, viewed from aisle 0 towards the positive *x*-direction.

A total of 1265 nodes based on TEXAS INSTRUMENTS CC2510 transceiver chips was randomly distributed at different heights on the fronts of 71 shelves (1 m width) as wideband localization targets. One shelf is shown exemplarily in Figure 3b, a closeup of the ESL nodes in Figure 3c. As the regular operation of the store was not to be impaired by the measurements, it was impossible to place the nodes arbitrarily. The exact number of deployed target nodes per shelf is indicated in Figure 2a. The nodes were mounted in addition to the regular ESLs actually used as price tags in this store. Due to the geometry of the shelves, an uninterrupted line-of-sight (LoS) between nodes and anchors was only available within the same aisle. The hardware of the target nodes and the regular ESLs displaying product prices was identical. The only difference was a modified firmware which enabled the wideband transmission scheme as described in Section 2.1.

#### 3.1.2. Anchor Placement

Receiving anchors were mounted in the three aisles on top of the shelves at a height of z=2.6m. Similar to the situation for the nodes, the anchor placement was constrained by the given structures (shelves, etc.) and an uninterrupted store operation. The fixed height of the anchors above the nodes leads to a low positioning precision in vertical direction. This is critical mainly for nodes on shelves close to anchors, where the vertical component contributes significantly to the absolute Euclidean distance between the nodes and this anchor. However, as shown below in the anchor placement discussion, this effect turns out to be tolerable.

**Figure 4:**
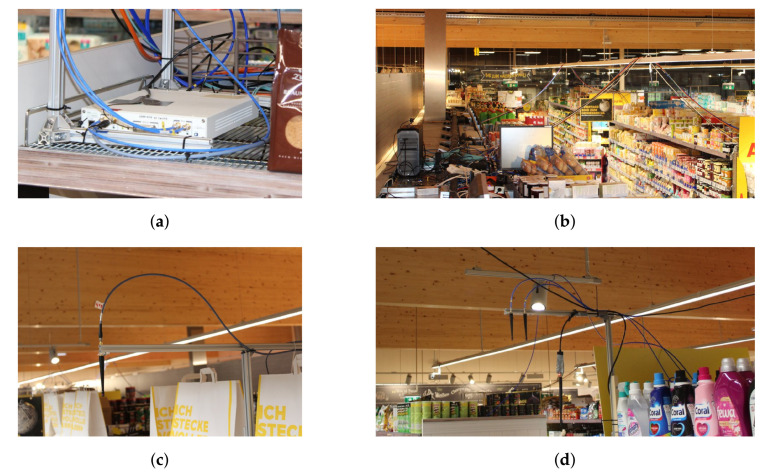
Measurement setup components mounted in the test environment. (**a**) USRP #2 mounted on the top of a shelf. (**b**) Overview over the covered area. Visible are the host setup, the nodes on the shelves of aisle 1, and the cable harnesses to USRP #2 (foreground) and #3 (background). (**c**) Anchor 3. (**d**) Anchor 5.

Anchors are visible in Figure 3a and Figure 4c,d. Different distances between the anchors and different single and dual-antenna anchor configurations allow to assess the consequences on the obtained positioning results as undertaken in Section 4.2.

As each antenna of an anchor requires a dedicated physical receiver channel, the number of anchors was limited by the available channels of the used radios (twelve). In all following floorplan figures, the anchors are marked as circles for single-antenna anchors (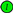), representing their omni-directional characteristics and as diamonds for dual-antenna anchors (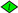), indicating the array boresight with best AoA resolution by pointing in aisle direction (symmetrically). In accordance with the used positioning error bound framework [21], the anchors are labeled l=0,⋯,7. This framework was used to evaluate the PEB for the entire scenario, shown in Figure 2b. To do so, certain channel parameters have to be assumed, such as propagation loss, power delay profile (PDP), receiver noise figure, additional attenuation for non-LoS links, etc. These parameters were chosen from the literature [9,15,18], to resemble the considered scenario. Naturally, this best guess approach is suboptimal and deviations of the measurements from these theoretical results must be anticipated. Nonetheless, this approach gives a basic idea of possible consequences of certain anchor placements and was used extensively to analyze possible schemes for the given scenario prior to the measurement campaign. As the position estimator used in this work itself does not rely on these coarsely assumed channel parameters, no negative impact of inaccurately chosen parameters must be anticipated.

It is possible, in principle, to conduct a detailed analysis of the true channel parameters from the obtained data set and even include them into the estimator. This is, however, considered out of scope of this paper.

### 3.2. Measurement Setup

**Figure 5:**
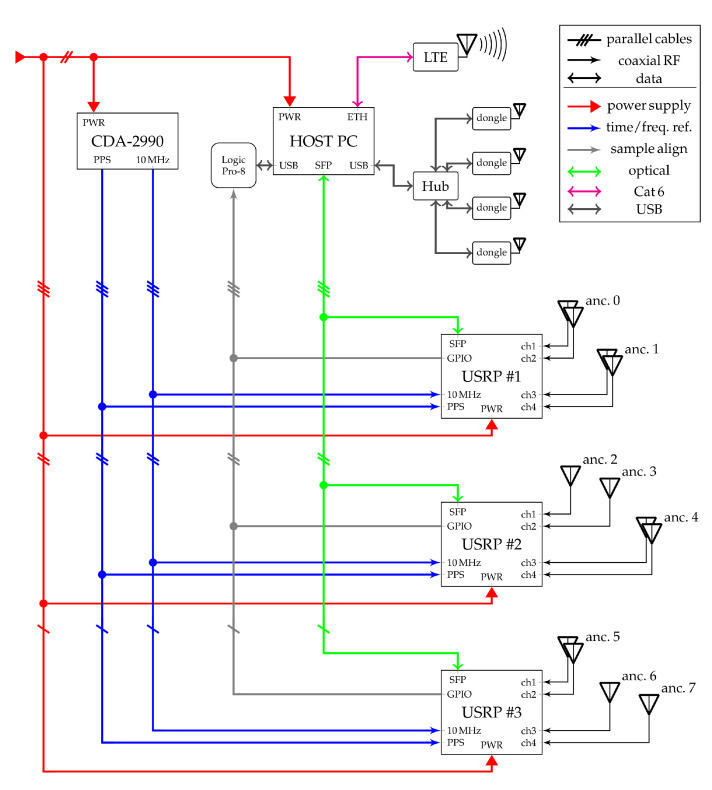
Block diagram of the full measurement setup. The USB dongles used to communicate with the target nodes are connected via a USB hub. The power supply uses a power cord with 230 V AC. All RF cables are 50 Ω coaxial cables with SMA connectors. The connections indicated as parallel use distinct cables for each USRP radio platform, connected to distinct ports at the host setup, e.g., three SMA connectors for the 10 MHz reference, three SFP fiber connectors, etc.

The used hardware, its deployment as wideband receiver infrastructure, as well as synchronization requirements are described in the following. The locations of all components in the scenario are indicated in Figure 2c. A full block diagram of the setup is shown in Figure 5.

#### 3.2.1. Receiver Hardware

The wideband receiver infrastructure was based on three ETTUS RESEARCH/NI USRP SDR platforms as seen in Figure 4a, each consisting of X310 motherboards with two TwinRX daughterboards. The three USRPs were placed on the top of shelves at the location in indicated in Figure 2c as (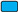). With four physical receiver channels each, these receivers provide a total of twelve physical channels. The sampling rate allows to cover the full ISM band at 2.4 GHz. Further technical details on these receivers can be found in the product documentation (https://kb.ettus.com/X300/X310, https://kb.ettus.com/TwinRX; both accessed on 1 February 2022).

The USRP platforms were connected to a host setup comprising a PC(HP Z4 workstation, Intel XeonW-2123 CPU, 64 GB RAM) for configuration and data acquisition, as well as a synchronization source. Details on the synchronization of the system are stated in Section 3.2.3. The host setup was placed on a shelf at a central location as seen in the wide-angle view of the scenario Figure 4b. Each USRP was connected via a cable harness comprising an AC power supply, an optical fiber cable (Duplex LC/LC 50/125 μm OM3, 20 m and SFP+ connectors) for data exchange, and RF cabling for synchronization. Further, USB dongles (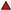) were used for node configuration using a standard communication protocol. All cables and the dongles were fixed to the suspended lighting installation of the store.

Configuration of the USRPs was performed using the open source multi_usrp API framework of the ETTUS RESEARCH/NI USRP Hardware Driver (UHD) UHD-3.15.LTS (https://github.com/EttusResearch/uhd; accessed on 28 October 2021). The provided field-programmable gate array (FPGA) gateware allows only live streaming of the data. However, the high amount of rawdata generated by the receivers (4.8 GB/s) poses a challenge to the host PC acting as data sink. Thus, a customized adaption of the gateware was used which supports buffering of a limited time recording in the X310 RAM and forwarding to the host at a lower rate. Details on this gateware can be found in [32] (chp. 5).

The pre-processing of the wideband signals was performed right after recording on the host PC. The full measurement process for one node, including waiting for the standardized wake-up times of the node, self-calibration, wideband transmission, recording, pre-processing, and data upload, took about 60 s in this implementation. The process was not optimized for speed.

In order to be independent of the on-site corporate network and provide as much flexibility as possible, an LTE access point was used to connect to a mobile network for data upload and remote measurement control.

#### 3.2.2. Anchor Configuration

The total of twelve synchronized physical channels of the three X310+TwinRX USRP units were grouped into (logical) receiver anchors. Two variants were deployed, either with one or two antennas. Accordingly, single-antenna anchors allow only for TDoA positioning, while dual-antenna anchors provide additional AoA information. With a given number of available physical channels this means a trade-off between number of logical anchors and array sizes. The used mixture allowed for testing the influence of different anchor configurations and placements.

Half-wave antennas (LINX ANT-2.4-CW-QW https://www.mouser.at/datasheet/2/238/ant-2.4-cw-qw-1659137.pdf; accessed on 21 February 2022) pointing downwards were used for the logical anchors. The radiation pattern of a single antenna is uniform in the horizontal plane, i.e., for all azimuth angles. In the case of dual-antenna anchors, the antenna spacing was 6 cm which is slightly below a half-wavelength for the used 2.4 GHz ISM band. The boresight of these arrays was oriented in the direction of the respective aisles to yield the best angular resolution. The single and dual-antenna anchor configurations can be seen in Figure 4c,d, respectively. The vertical polarization of the used antennas was in accordance with the vertically polarized antennas of the transmitting nodes.

#### 3.2.3. Synchronization and Calibration

The used ETTUS RESEARCH/NI X310 SDR platforms support synchronization via a 10 MHz frequency reference signal and a pulse-per-second (PPS) signal over cable connections. A free-running ETTUS RESEARCH/NI OctoClock CDA-2990 synchronization source was used to provide both the required 10 MHz and PPS signals, as the given indoor environment made it impossible to use global navigation satellite system (GNSS) time-synchronization. Six 15 m cables (MINI-CIRCUITS CBL-50FT-SMSM+) were used to forward the common frequency and time base to the three SDR platforms. Triggering of the wideband recording was performed via the UHD API upon a timestamp derived from the PPS signal.

Due to the characteristics of the USRPs, the used time synchronization method sometimes produces glitches resulting in a misalignment between the three units by plus/minus one sample. As a quick fix, identical feedback cables were used to capture a rising edge on an X310 FPGA GPIO generated upon the start of sampling by each of the three devices. The capturing was performed at the host setup for all three SDR platforms using a USB logic analyzer (SALEAE Logic Pro-8). This procedure allowed for exact time-synchronization of the used USRP platforms.

The time-offsets between logical anchors due to different cable lengths between antennas and USRP physical channel radio front-ends were pre-measured. Similarly, a calibration was performed for the phase-offsets between the channels of dual-antenna anchors. This is required for correct TDoA and AoA measurements, respectively, as addressed in detail in [21]. A hand-held signal generator(SIGNAL HOUND VSG60A) and a characterized signal splitter(MINI-CIRCUITS ZN2PD2-63-S+) were used to feed a known wideband signal to the receivers via the antenna ports instead of the actual antennas. This was performed between all logical anchors for group delay difference calibration and between the channels of the dual-antenna anchors for phase difference calibration. Simultaneously, the power levels and the frequency response of the radio front-ends was calibrated.

The IQ-imbalance is no issue for the used receiver hardware as sampling is performed at a low intermediate frequency (IF). Phase alignment between different logical anchors was not required as meaningful AoA information cannot be deduced from such a constellation. A possible frequency misalignment of the anchors was assumed to be negligible because of the used 10 MHz reference frequency distribution.

## 4. Measurement Evaluation

The uploaded equivalent channel impulse response of each node, as described in Section 2.1, was used to run a simple MLE positioning algorithm as described in the following. (Vectors and matrices are denoted by bold letters, unlike scalars.) It is based on a system model with an assumed circularly-symmetric complex normal distribution of the sampled baseband data vectors $r_{m}^{\left(l\right)}$, received at the *m*-th antenna of the *l*-th anchor,

(1) $$r_{m}^{\left(l\right)}∼\mathrm{CN}\left(\alpha^{\left(l\right)}s_{m}^{\left(l\right)},C_{m}^{\left(l\right)}\right),$$

with a mean vector $\alpha^{\left(l\right)}s_{m}^{\left(l\right)}$ and a covariance matrix $C_{m}^{\left(l\right)}$. The mean vector consists of the signal vector $s_{m}^{\left(l\right)}$, a version of the sampled transmitted wideband signal, shifted in time by the LoS ToF to the *m*-th antenna of the *l*-th anchor, multiplied with the scalar complex-valued magnitude $\alpha^{\left(l\right)}$ for the LoS channel between transmitting node and *l*-th anchor. For the sake of simplicity and to allow faster (real-time) processing of the obtained data, the DMC as additive random process described in [18] was not included. Thus, the covariance matrix is diagonal with the additive white Gaussian noise (AWGN) variance $\sigma_{w}^{2}$ on the main diagonal, i.e., $C_{m}^{\left(l\right)}=\sigma_{w}^{2}I$. With this simplification, the probability density function for Equation (1) can be described as

(2) $$p_{r_{m}^{\left(l\right)}}\left(r_{m}^{\left(l\right)};\theta \right)\propto \exp\left(-\frac{1}{\sigma_{w}^{2}}{∥r_{m}^{\left(l\right)}-\alpha^{\left(l\right)}s_{m}^{\left(l\right)}∥}^{2}\right).$$

It is parameterized on the vector θ, which summarizes all unknown parameters. These comprise the true transmitter position as Cartesian coordinates and the time of transmission (ToT) of the wideband signal, which is required due to the lack of synchronization between node and receiver infrastructure. The parameter vector further contains the LoS magnitudes $\alpha^{\left(l\right)}$ for all *L* anchors as nuisance parameters. Under the assumption of statistically independent AWGN with identical variance for all receiver channels, the MLE for the parameter vector θ can be formulated as

(3) $$\hat{\theta }=\arg\max_{\theta }\left\{\prod_{l=0}^{L-1}\prod_{m=0}^{M^{\left(l\right)}-1}p_{r_{m}^{\left(l\right)}}\left(r_{m}^{\left(l\right)};\theta \right)\right\},$$

using the joint likelihood function of all data. Due to the assumption of statistically independent noise, it is simply the product of the probability density functions for the $M^{\left(l\right)}$ antennas of the *l*-th anchor and all *L* anchors.

For the evaluation of the estimator in the given scenario, the (vertical spatial) *z* component was omitted as discussed in Section 3.1.2. A grid search was performed over the remaining parameters, mainly the *x* and *y* coordinates, as well as the individual ToT, due to lack of synchronization between node and receiver infrastructure. The resolution was 40 cm for both spatial and the temporal components.

Even though this AWGN-only approach is suboptimal in the given indoor scenario due to the DMC of the channel, it is sufficient for a proof of concept of the introduced localization technique. It should be noted that, as a consequence of the used estimator ignoring the DMC, the PEB is not expected to be attained generally but only in cases where DMC is not dominant. The following bound evaluations are thus to be seen as a qualitative, rather than an exact analysis. It still allows for a rough assessment which areas of the scenario will work better or worse and a coarse prediction of the expected error distribution. A detailed analysis of the exact channel parameters and optimum positioning algorithms is beyond the scope of this paper and left for future work on this topic.

### 4.1. Overview

**Figure 6:**
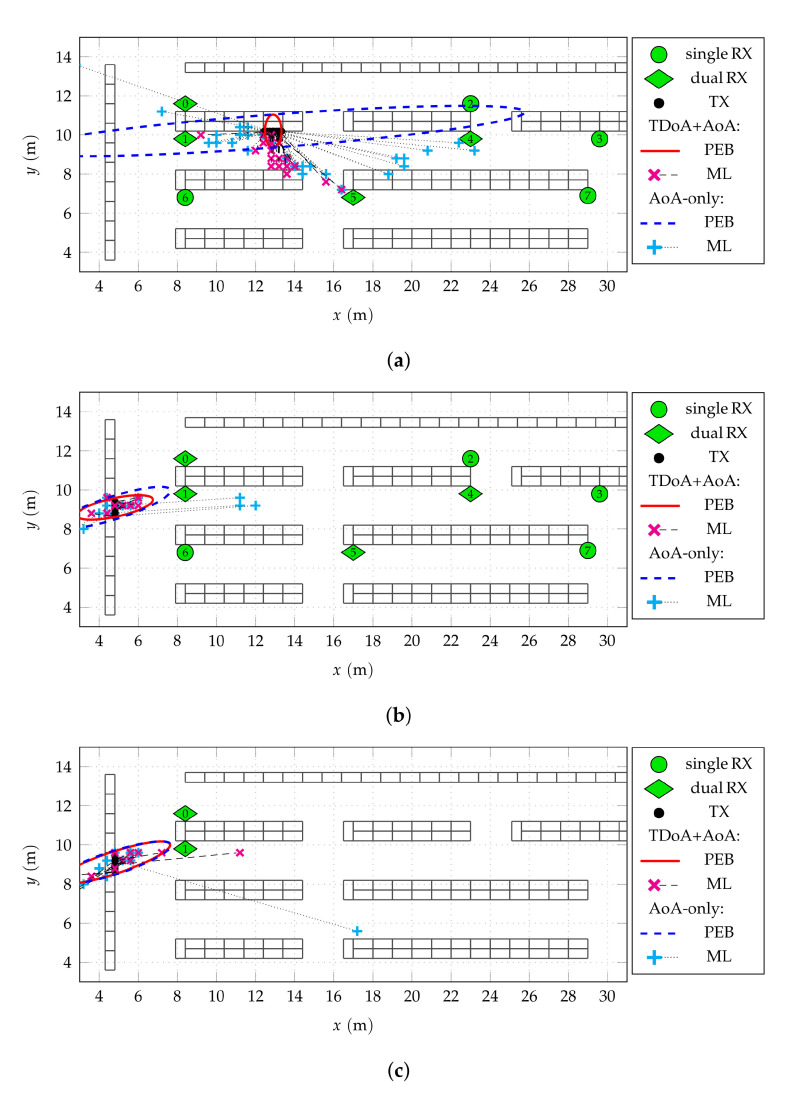
Position estimates for selected shelves and different receiving anchor configurations. Both TDoA+AoA as well as AoA-only estimates are shown. The 99% position error bound (PEB) ellipses are indicated for both methods. The spatial resolution is 40 cm. (**a**) Shelf with 29 nodes in aisle 2 and all anchors active. (**b**) Shelf with 14 nodes in aisle 0 and all anchors active. (**c**) The same shelf with with only anchors l=0,1 active.

For illustrative purposes, Figure 6 shows the obtained position estimates for the nodes on selected shelves. Assuming independent measurements for multiple nodes on the 1 m wide shelves allows for a comparison with the theoretical 99% PEB. The topic of receiving anchor synchronization is addressed exemplarily by not only evaluating the position estimate for what is assumed to be perfectly synchronized anchors, but also the AoA-only case. Thus, without sufficient time-synchronization, single-antenna receivers do not contribute any information about the node position. The AoA-only PEB ellipses are shown accordingly.

In Figure 6a, a shelf in the center of the covered area with 29 transmitting nodes (•) is shown. The position estimates using both TDoA and AoA information (×- -) show a bias towards the middle of the aisle with a median absolute error of 1.39 m. The associated PEB ellipse (—) slightly underestimates the spread of the actual position estimates, which is likely due to neglecting the DMC. Whereas this accuracy is not sufficient to find a single node, the location within the aisle and even identification of the correct shelf is possible for many nodes. Assuming a lack of time synchronization between the anchors results in AoA-only estimates (+· · ·). Again, a bias towards the middle of the aisle is observed while the spread in *x* direction is within the according bound (- - -). Even though the median error in this case is as high as 3.18 m, the identification of the correct aisle is still possible.

For certain geometrical arrangements of nodes and anchors, the TDoA information does not contribute significantly. For example, the lateral coverage of aisle 0 allows for a certain positioning quality even without time synchronization of the anchors as visible in Figure 6b. Besides a few outliers, the position estimates lie within the region predicted by the PEB evaluation for both the synchronized as well as the AoA-only case. This indicates rather weak DMC in this wider aisle compared to the situation for shelves in the narrower aisle 2 shown in Figure 6a. Comparing these results with an evaluation using only anchors l=0,1 only, as shown in Figure 6c, it becomes obvious that more or less the entire position information stems from these two anchors.

**Figure 7:**
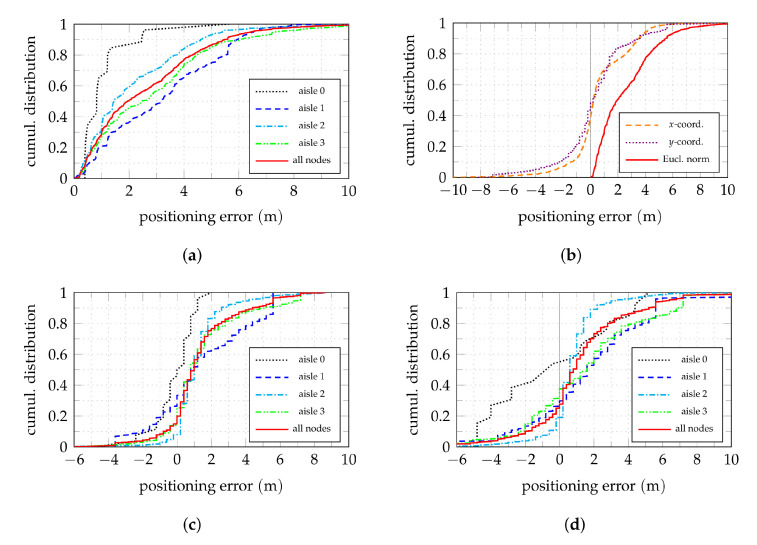
Cumulative positioning error distributions in the horizontal plane using both TDoA and AoA information. In (**a**–**c**), all anchors are active. (**a**) Shows the Euclidean error norm for all nodes and broken down by aisle. (**b**) Compares the Euclidean to its *x* and *y* components. (**c**,**d**) show the error component orthogonal to the shelf surface for each node. Positive values are distance from the shelf surface towards the middle of the aisle. (**c**) All anchors active. (**d**) Anchors l=1,3,4 active.

Figure 7 shows cumulative distributions of the positioning error in the horizontal plane. The distribution of the Euclidean error norm for all nodes is shown in Figure 7a (red), together with the distributions for the nodes within their respective aisles. The stepped appearance in the curves are due to the evaluated spatial grid and the comparatively low number of nodes in the case of aisle 0. With the used localization algorithm, about 50% of the nodes have an absolute positioning error below 2 m.

Considering the *x* and *y* coordinates of the errors separately instead of the Euclidean norm for all nodes as shown in Figure 7b shows a slight non-symmetric behavior. This can be explained by the asymmetric anchor configurations within the aisles, as well as the special coverage of aisle 0. For most nodes, the localization in *x*-direction works slightly better.

In the light of a possible goal of aisle detection, yet another aspect is important. The previous claim of a typical bias of the position estimates towards the middle of the aisle can be confirmed when examining only the error component orthogonal to the respective shelf front for each node as shown in Figure 7c. In this case, the positive direction is always counted away from the shelf towards the middle of the aisle, i.e., positive values correspond to position estimates that lie in front, negative values to estimates within or behind of a shelf. For positive errors below 2.5 m, i.e., the width of the aisles including the shelf on the opposite side with a depth of 0.5 m, this means that the correct aisle could be detected. Below −0.5 m and above 2.5 m, the position estimates lie in wrong aisles. (Even though aisle 0 is a special case as the dimension and geometry of the aisle and its neighbors are different from aisle 1 to 3, the discussed idea applies as well.) This means that correct aisle detection is possible for about 70% of the nodes. The values, however, differ for the four considered aisles. For example, aisle 0 with a larger width and a fundamentally different kind of coverage shows a significantly smaller spread than the other aisles. Among the three parallel aisles 1 to 3, aisle 2 has (by far) the best performance with a correct aisle detection of about 90%. One might be tempted to assume that the anchors in the adjacent aisles contribute to this effect. Evaluating, however, the position estimator using only the signal received by the anchors within aisle 2, i.e., anchors l=1,3,4 active, as done in Figure 7d shows that the outcome for this aisle is hardly affected. On the one hand, it can be concluded that the influence of anchors in adjacent aisles, and thus also time-synchronization of anchors in adjacent aisles, is uncritical for this kind of evaluation. On the other hand, this highlights the importance of anchor layout and placement, as this is the major difference between the three aisles. Thus, the influence of anchor placement is studied further in the following section.

### 4.2. Anchor Placement Analysis

A major question for the implementation of an actual receiver infrastructure is the placement of anchors for a sufficient coverage of a given scenario. For the measurement campaign introduced here, this placement was chosen such that a comparison of different arrangements is possible. In each of the three parallel aisles 1 to 3, different amounts of both single and dual-antenna anchors in different arrangements along the aisles were deployed, as seen in Figure 2c. Aisle 0 was covered in a fundamentally different way by re-using the lateral anchors l=0,1,6.

In order to allow for a descriptive illustration of the results, it is beneficial to group the nodes shelf-wise. This allows to assess the achievable positioning quality for different areas of the covered scenario. The median of the positioning error of all nodes on a shelf can serve as an ad hoc measure for this. As already highlighted in Section 3.1.1 and seen in Figure 2a, some shelves could not be equipped with a sufficient number of nodes due to the constraints given by the uninterrupted store operation. This means that the error median is less reliable for those shelves. Therefore, one should focus on shelves with more than some minimum number of nodes in the following. (Twelve was empirically found to be an amount of nodes for which the positioning error median does not significantly depend on the selection any more when drawn from all nodes of a shelf with more nodes.)

**Figure 8:**
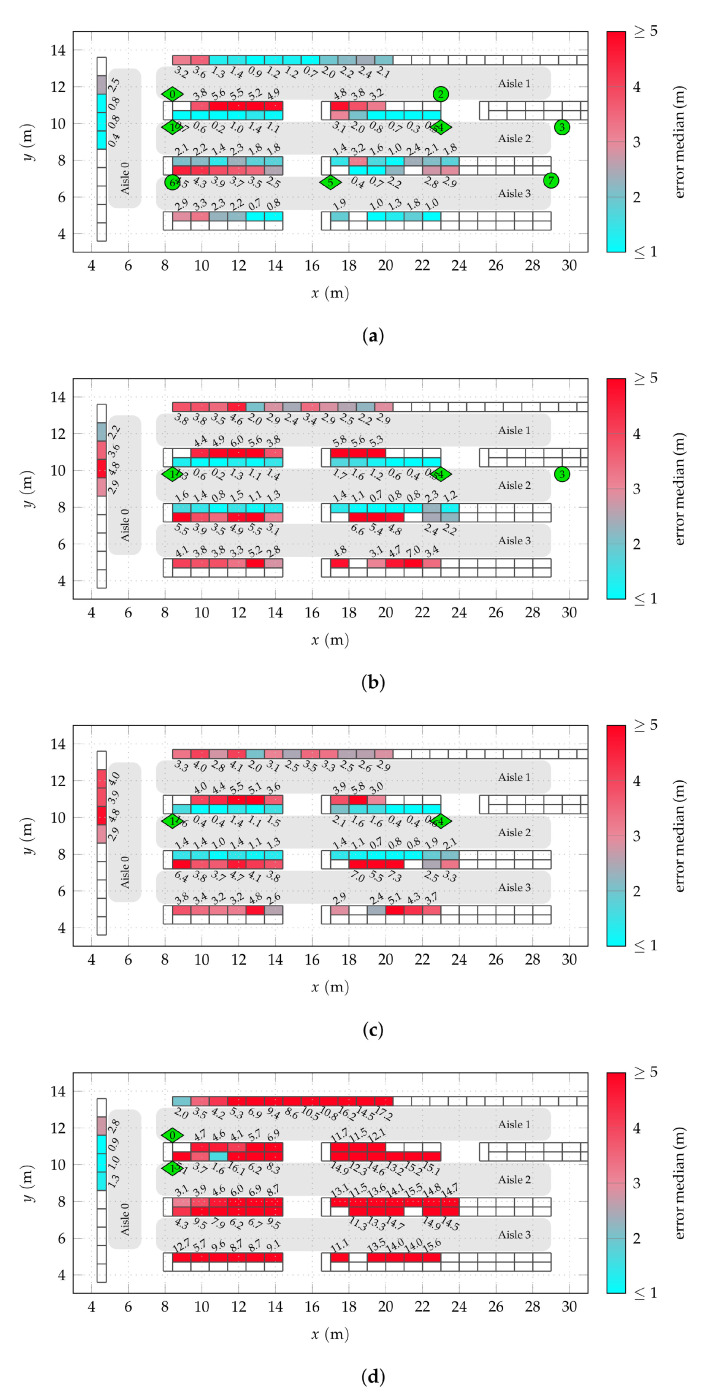
Shelf-wise median of the absolute positioning error for different anchor configurations. (**a**) All anchors active. (**b**) Anchors l=1,3,4 active. (**c**) Anchors l=1,4 active. (**d**) Anchors l=0,1 active. (The one “good” shelf in aisle 2 has only 1 node and must thus be considered an outlier).

**Figure 9:**
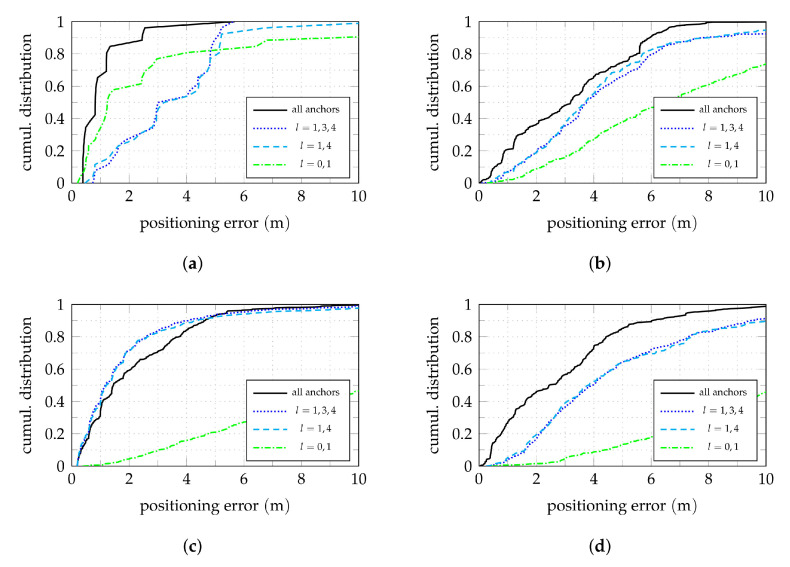
Cumulative absolute error distributions for the four aisles. The anchor configurations used in Figure 8 are shown. Note that in the previous shelf-wise consideration shelves with a high number of nodes do not stand out in particular, while here all of them contribute individually. The curves for all anchors are the same as in Figure 7a. (**a**) Aisle 0. (**b**) Aisle 1. (**c**) Aisle 2. (**d**) Aisle 3.

Figure 8 shows the median of the absolute positioning error by shelf using the data received by various combinations of active anchors. The associated cumulative distribution of the positioning error for all nodes are broken down for the four covered aisles in Figure 9 for the same active anchor combinations.

Apart from the special case of aisle 0, aisle 2 has the best coverage, which can also be seen in Figure 7a. As previously claimed, the localization performance in an aisle is more or less independent of the coverage of adjacent aisles. This can be confirmed by comparing Figure 8a to Figure 8b. As for the aisle detection, Figure 7c,d, the median errors for aisle 2 do not deteriorate when removing the anchors from aisle 1 and 3. For some shelves (and nodes) in this aisle, the performance is even improved, which can also be seen in Figure 7a, especially for errors from 2 m to 4 m. Even though this effect is rather weak, it indicates that including prior information on the scenario geometry, such as information on the possible LoS and non-LoS links could be advantageous.

Compared to aisle 2, aisle 1 and 3 exhibit significantly worse performance for the shelves close to the LoS from anchor 0 to 2 and 5 to 6. Considering the previous reasoning of adjacent aisle anchors having a minor effect on the performance, a possible explanation is the reduced available AoA information. For one thing, even tough the direct LoS between nodes on the close sides of the aisles is unobstructed, it runs close to the surface of the shelves. This means that, due to the metallic shelf structure, the assumption of free-space propagation does not hold. Additionally, in contrast to anchors 1 and 4 in aisle 2, the anchors 2, 6, and 7 are single-antenna anchors which do not contribute AoA information.

In these cases of limited AoA information, the consequences of the LoS link being close to the shelf surfaces becomes even more obvious. The anchors were not mounted in the middle of the aisles for structural reasons, but rather 60 cm off-center, towards one side of the aisles, as visible in the floorplans. For these close sides of the aisles, the performance is significantly worse than for the opposite sides in both aisles 1 and 3.

By neglecting the *z* coordinate for processing, potential errors of up to 2 m can be caused, as this is the maximum vertical distance between nodes and anchors for shelves beneath an anchor. This bias should decrease for a greater horizontal distance from the anchors where the vertical component has a lesser share of the direct LoS link. Even though such an effect can be observed especially in aisles 1 and 3, the fact that it does not occur in aisle 2 hints towards the reduced AoA information available in these aisles as a reason. Especially, as even the dual-antenna anchors do not provide good angle resolution in off-boresight direction. In the work at hand, this effect was assumed to be tolerable, as including it into the evaluation would require more involved anchor antenna arrangements, possibly a phased array antenna [33], which was unfeasible with the available receiver hardware.

The additional single-antenna anchor 3 providing some additional TDoA information was expected to improve positioning, especially in regions lying in the directions of least obtained angular information (orthogonal to array boresight) of one of the two dual antenna anchors 1 and 4. In such regions with nodes below the arrays, the gain of the used antennas is low and, due to the large inclination angle, neglecting the vertical coordinate alone causes a potential error of up to 2 m (the vertical distance between nodes and anchor). Comparing Figure 8b to Figure 8c, however, shows that no significant improvement can be achieved. Thus, even though for some parts of aisle 1 and 3, especially when being geometrically advantageous, single-antenna anchors might be enough for a sufficient performance, comprehensive coverage of an aisle can be provided by two dual-antenna anchors at its ends with array boresight facing along the aisle, as done in aisle 2. The distance of 14 m between the anchors is still short enough to allow full coverage.

Reconsidering the exemplary shelf evaluation Figure 6c for aisle 0, Figure 8d confirms the statement that in aisle 0 the most the available information is provided by anchors 0 and 1. Thus, in such a special case of aisle coverage via dual-antenna anchors in other (orthogonal) aisles can be sufficient. However, as seen in Figure 9a, the number of outliers increases. Furthermore, it must be expected that for regions far off the boresight of the anchors, e.g., the shelves in the lower region of aisle 0, not equipped with nodes in this case, performance will deteriorate. This is due to the reduced angle resolution of the antenna arrays in such directions [34] (sct. 3.11) and the acute angle between the LoS connections from the node to the two anchors, being disadvantageous for triangulation. With a hypothetical dual-antenna anchor coverage of aisle 3, this issue could likely be resolved.

### 4.3. Clustering

Under the given constraints of reusing existing hardware in a difficult environment, the possibilities for precise localization are limited. A natural approach is, thus, the use of multiple different information sources. As previously mentioned, commonly used transceiver chip used in such environments support node-to-node RSS measurements. Such, typically narrowband, methods are prone to multipath propagation effects, and thus of limited use for localization. A simple way to combine RSS measurements with the information obtained by the introduced wideband localization scheme is to find a cluster of potential spatially close neighbors based on the RSS measurements and use the combined wideband information. For this, the wideband position estimates of multiple ($N_{\mathrm{cl}}$) neighbors of one node are used to calculate the mean as a new estimate for said node. The members of the cluster for a certain node can be found for example by selecting the neighboring nodes with strongest RSS.

In this way, no clustering of the RSS measurements into categories is required. This selection approach is not ideal due to the expected non-ideal relation between distance and RSS measurements, caused by multipath fading. To evaluate the usability, it is compared with a genie-aided method for which the $N_{\mathrm{cl}}$ spatially closest neighbors are selected as a cluster for a node, based on their position ground truth. This idealized selection process only makes use of the relative position of each other and can therefore be considered as realistic in the sense of using only information that could be obtained theoretically from RSS measurements under ideal conditions (no DMC). It represents a simple pathloss model, assuming a monotonic decline of the power received from other nodes over distance to the receiving node. In the given scenario, a severe attenuation of the received signals has to be assumed if shelves block the LoS link between nodes. Therefore, the described nearest neighbor selection approach is restricted to the aisle of an evaluated node. Possible multipath fading is neglected.

**Figure 10:**
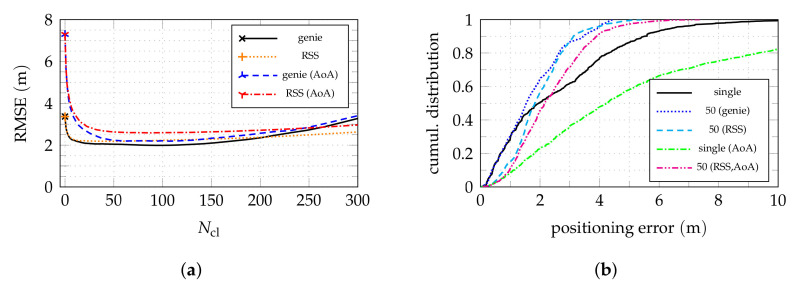
Effects of clustering on overall positioning root-mean-square error (RMSE) and error distribution for single nodes. A cluster size of $N_{\mathrm{cl}}=0$ (indicated by a marker) means only the node by itself. All anchors active. (**a**) RMSE over cluster size for genie-aided and RSS-based clustering. The TDoA+AoA (not explicitly indicated) and the AoA-only cases are compared. (**b**) Cumulative absolute error distribution for a single node and one fixed cluster size of $N_{\mathrm{cl}}=50$.

Repeating these procedures to obtain cluster estimates for all nodes yields the following results. As a measure for overall positioning performance, the root-mean-square error (RMSE) of all position estimates is evaluated. Figure 10a shows this RMSE for both genie-aided and RSS-based chosen clusters, using the wideband position estimates obtained with full anchor synchronization (using both TDoA and AoA information), as well as only AoA information. The marked cluster size $N_{\mathrm{cl}}=0$ is equivalent to the raw position estimates without clustering. Using the full information, this initial RMSE is 3.36 m, for AoA-only 7.29 m. The overall RMSE can be reduced strongly, even for small cluster sizes. With $N_{\mathrm{cl}}=50$ and measured RSS data, the RMSE for full anchor synchronization is reduced by a factor of 1.53 to 2.19 m, for AoA-only, even by a factor of 2.77 to 2.63 m. The difference to the genie-aided selected clusters of the same size is only 14 cm and 40 cm, respectively. This indicates that the idealized genie-aided cluster selection models the given situation well.

For $N_{\mathrm{cl}}>50$, no significant improvement can be gained anymore. If the cluster size is chosen too large, even a degradation can be observed. This can be explained by the number of nodes available for the cluster. If the selected nodes already have a very weak RSS or are too far from the cluster center, no additional gain is achieved. Naturally, the observed effects depend on the actual scenario. For example, if the node placement is to sparse (as seen later in the case of aisle 0), the effects of a high $N_{\mathrm{cl}}$ kick in earlier as more nodes will be at a larger distance. In the considered scenario, the median distance between a node and the nodes in its cluster for $N_{\mathrm{cl}}=50$ is 1.03 m for genie-aided and 2.25 m for RSS-based clustering on average. In both cases, this means that about the half of the cluster nodes are typically within a distance of the aisle width or shelf height. This is in accordance with the intuitive notion of clustering of nodes on a shelf or within a section of the aisle.

The associated cumulative positioning error distributions for a single node ($N_{\mathrm{cl}}=0$) and $N_{\mathrm{cl}}=50$ are shown in Figure 10b. With both full anchor synchronization and AoA-only, the clustering mainly has the potential of reducing the amount of severe outliers and smoothing the curve due to the averaging effect. While the genie-aided clustering has a positive effect for all nodes, with real RSS data a possible degradation for some nodes with initially good results has to be anticipated. The median absolute error is only reduced significantly in the case of AoA information only, from 4.23 m to 2.15 m. If the TDoA information is included, it is only reduced from 1.94 m to 1.82 m.

**Figure 11:**
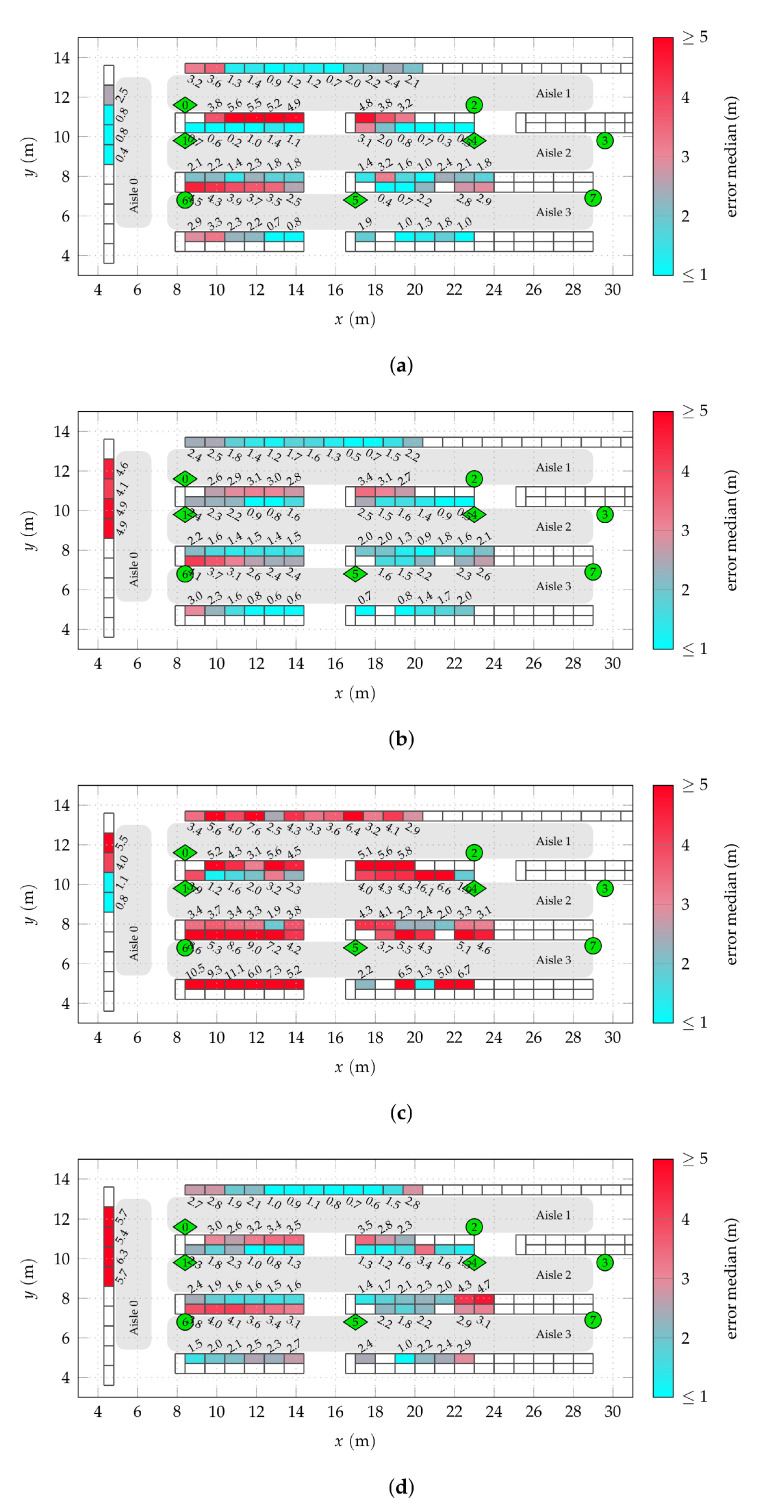
Shelf-wise median of the absolute positioning error for the described clustering approaches. In (**a**) and (**b**), both the TDoA and AoA information is used, in (**c**) and (**d**) only the AoA information. The clustering is based on actual node-to-node RSS measurements. (**a**) No clustering, TDoA+AoA. (**b**) $N_{\mathrm{cl}}=50$, TDoA+AoA. (**c**) No clustering, AoA-only. (**d**) $N_{\mathrm{cl}}=50$, AoA-only.

**Figure 12:**
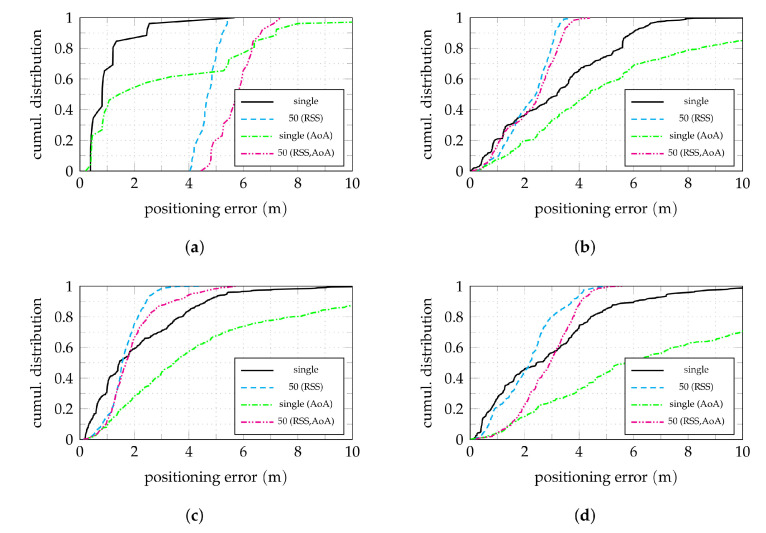
Cumulative absolute error distributions for effects of different clustering approaches in the four aisles. A number of $N_{\mathrm{cl}}=50$ nodes is used for clustering. All anchors active. The TDoA+AoA (not explicitly indicated) and the AoA-only cases are compared. The curves for single nodes are the same as in Figure 7a. (**a**) Aisle 0. (**b**) Aisle 1. (**c**) Aisle 2. (**d**) Aisle 3.

The median of the absolute positioning error by shelf, shown in Figure 11, allows to analyze the effects of RSS-based clustering depending on the anchor configurations and aisle geometry, similar to Figure 8. An accompanying break down by aisle is shown in Figure 12. Comparing the results with full anchor synchronization (TDoA+AoA) for single nodes without clustering (Figure 11a) with clustering using $N_{\mathrm{cl}}=50$ (Figure 11b) again shows the averaging effect. The situation for shelves with a higher initial error is generally improved, while for some previously well-working shelves the error increases. An exception is aisle 0, for which clustering worsens the achievable error. The cumulative error shown in Figure 12a confirms this result. This clearly shows the limitations of the proposed clustering approach. If an insufficient number of neighbors is available in the immediate vicinity of a node, a fixed cluster size is inadequate. With only 27 nodes in aisle 0, $N_{\mathrm{cl}}=50$ leads to the selection of nodes in other aisles with position estimates far off aisle 0. The implementation of an RSS threshold to cap the number of used nodes might help to resolve this issue.

In the case of only AoA information available for localization, the effect of clustering is even more striking. While using the raw position estimates as performed for Figure 11c does not perform well anywhere in the examined scenario, clustering greatly improves the situation almost everywhere (apart from aisle 0). This can be explained by reconsidering the distribution of the individual position estimates as shown in Figure 6a. While individual results of AoA-only position estimation may be off by more than ten meters, all results are scattered with a mean close to the actual shelf. The clustering proposed here makes use of this effect for every individual node.

## 5. Conclusions

In this paper, we demonstrated the usability of a customized wideband modulation scheme on narrowband IoT transceiver chips for indoor localization. This wideband signal generation, enabled by a mere firmware update, was successfully tested with more than 1200 blindly transmitting nodes under real-world conditions in an operating grocery store. A receiver infrastructure based on USRP SDR platforms was used to capture the transmitted waveforms. By using AWGN-only MLE for the transmitter positions, the achieved performance of the proposed localization scheme was assessed. Different receiver anchor arrangements were compared, showing the high benefit of the joint use of both TDoA and AoA information.

Thus, good coverage of aisles can be achieved using dual-antenna at their ends. Neglecting the vertical dimension for the positioning algorithm has only a minor impact in this case. Thus, comparatively complex anchor antenna arrangements allowing better vertical positioning precision are unlikely to yield major improvements.

The influence of the aisle structures in the scenario was evaluated. A general bias towards the middle of the aisles was observed. Further, an attempt for fusing the TDoA and AoA information obtained by the wideband node-to-infrastructure measurements with node-to-node RSS measurements was performed. This shows that the amount of positioning error outliers can be greatly reduced by simple RSS-based clustering, bringing the overall RMSE down to 2.19 m. Even when using only AoA information, this effect can be observed, indicating, that time synchronization of all anchors can be unnecessary in certain scenarios.

## 6. Outlook

Having successfully shown that the proposed method allows localization of blindly transmitting nodes under real-world conditions, long-term measurements under controlled conditions are currently ongoing. In the course of this continued campaign in a non-operating test store, several aspects will be investigated.

On the one hand, the receiver infrastructure solution used in this work is the rather expensive in terms of costs and required cabling effort. Pursuing a more efficient solution using less potent and cheaper receiver hardware, as well as anchor synchronization only where needed is the central part of the future work on this topic.

On the other hand, in order to allow for a wider field of applications, wideband signal generation on different narrowband transceiver hardware is being tested.

The application of a more sophisticated positioning algorithm, also taking into account DMC and possibly prior scenario geometry information, e.g., for ray-tracing as shown, for example, in [35], is likely to further improve the obtained results. An extension of the model to the third spatial (vertical) dimension might lead to improvement. This, however, would require the study of different anchor antenna arrangements as the currently used configuration does not provide information in this regard. The identified shortcoming of the proposed clustering method in the case of a limited amount of available neighboring nodes might be overcome by a smarter clustering algorithm, possibly allowing a dynamic cluster size selection.

The use of a Gaussian Mixture Model (GMM) is a promising approach, for example to discriminate between LoS and non-LoS links as shown, for example, in [36].

Tests in this regard are ongoing. The eventual goal is fusing of all available information sources such as RSS, TDoA, AoA, scenario geometry, etc. into a near-optimum joint position estimate.

## Data Availability

For non-commercial use in research, data sets are available on request.

## Abbreviations

The most important abbreviations used in the manuscript are listed below:

AoA	Angle of Arrival
CRLB	Cramér-Rao Lower Bound
CW	Continuous Wave
DMC	Dense Multipath Components
ESL	Electronic Shelf Label
FPGA	Field-Programmable Gate Array
IoT	Internet of Things
LoS	Line-of-Sight
MLE	Maximum Likelihood Estimation
PEB	Position Error Bound
PPS	Pulse-Per-Second
RMSE	Root-Mean-Square Error
RSS	Received Signal Strength
SDR	Software-Defined Radio
TDoA	Time Difference of Arrival
ToF	Time of Flight
UHD	USRP Hardware Driver
USRP	Universal Software Radio Peripheral
